# Effect of Compressive Stress on Copper Bonding Quality and Bonding Mechanisms in Advanced Packaging

**DOI:** 10.3390/ma17102236

**Published:** 2024-05-09

**Authors:** Tsan-Feng Lu, Ping-Yang Lee, YewChung Sermon Wu

**Affiliations:** Department of Materials Science and Engineering, National Yang Ming Chiao Tung University, Hsinchu 30010, Taiwan; s0881513.c@nycu.edu.tw (T.-F.L.); pyli922.c@nycu.edu.tw (P.-Y.L.)

**Keywords:** hybrid bonding, Cu–Cu direct bonding, surface creep, elastic deformation, void morphology, compressive stress

## Abstract

The thermal expansion behavior of Cu plays a critical role in the bonding mechanism of Cu/SiO_2_ hybrid joints. In this study, artificial voids, which were observed to evolve using a focused ion beam, were introduced at the bonded interfaces to investigate the influence of compressive stress on bonding quality and mechanisms at elevated temperatures of 250 °C and 300 °C. The evolution of interfacial voids serves as a key indicator for assessing bonding quality. We quantified the bonding fraction and void fraction to characterize the bonding interface and found a notable increase in the bonding fraction and a corresponding decrease in the void fraction with increasing compressive stress levels. This is primarily attributed to the Cu film exhibiting greater creep/elastic deformation under higher compressive stress conditions. Furthermore, these experimental findings are supported by the surface diffusion creep model. Therefore, our study confirms that compressive stress affects the Cu–Cu bonding interface, emphasizing the need to consider the depth of Cu joints during process design.

## 1. Introduction

Improving electronic chip performance is crucial, and heterogeneous integration stands out as a promising approach. Three-dimensional integrated circuit (3D IC) interconnection has emerged as an advanced packaging technology and has been extensively researched [1,2,3,4,5]. In the past, solder joints have been extensively employed as the primary interconnection method in 3D ICs [6]. In order to accommodate a greater number of input and output connections, it became necessary to decrease the size and spacing of the solder bumps. However, issues such as side wetting and bridge failure were encountered as the solder joints were scaled down [7,8]. These problems were overcome by hybrid bonding technology, which not only provides ultra-small joints and ultra-fine pitch, achieving a high number of inputs/outputs (I/Os) [9,10,11], but also has almost no thickness limitation [12,13]. This technology can significantly reduce the overall thickness in future 3D packaging, enabling the stacking of a large number of chips.

In hybrid bonding, the Cu surface undergoes chemical mechanical polishing (CMP), leading to the dishing effect, which is characterized by Cu dishing and SiO_2_ erosion during the over-polishing step [14,15]. The recess of Cu pads, which is typically a few nanometers [16], should be smaller than their expansions to ensure proper contact during annealing. This allows for oxide–oxide bonding to take place at room temperature before raising the temperature to approximately 250–300 °C for Cu–Cu bonding [17,18,19,20]. The requirement to elevate the temperature is attributed to the higher coefficient of thermal expansion (CTE) of Cu compared to the surrounding SiO_2_ [21]. At elevated temperatures, the Cu pad protrudes, establishing contact with the Cu pad on the opposite side, thus forming a permanent interconnection.

Achieving high-quality bonding requires two crucial prerequisites: precise CMP of Cu recess and sufficient expansion of Cu pads at bonding temperatures. It is essential for the recess of Cu pads to be smaller than their expansions to guarantee proper contact during annealing. Some numerical studies have shown that the expansion of Cu decreases as the dimensions of Cu pads are reduced. This may result in different compressive stress levels during Cu–Cu bonding.

In our previous studies, it was proposed that the thickness of the Cu film affects the quality of the bonding interface [22]. Additionally, the depth of copper joints has been widely suggested to affect the extent of Cu expansion [16,23,24]. However, direct confirmation of the effect of compressive stress on the bonding interface is still insufficient.

In this study, artificial voids were introduced at the bonded interfaces to investigate the influence of compressive stress on the bonding quality and bonding mechanisms at elevated temperatures of 250 °C and 300 °C, which are conditions widely studied in low-temperature Cu–Cu bonding. To facilitate a rapid and effective study of the Cu–Cu bonding interface, thin film structures have been widely employed in research, contributing significantly to the field of 3D ICs. Therefore, we adopted Cu thin films for our initial feasibility study. Through our research, we discovered that the magnitude of stress affects bonding quality, and considering the depth of Cu joints in chip design is imperative. 

## 2. Experimental

### 2.1. Cu Film Electrodeposition

The experimental setup involved the use of an electroplated Cu film on a Si wafer. Initially, a 50 nm layer of Ta was sputtered onto the Si wafer as an adhesion layer, followed by the deposition of a 200 nm thick Cu seed layer. The Si substrate was immersed in an electrolyte during the electroplating process. After completing the electroplating, the surface of the Cu film was flattened using chemical mechanical polishing (CMP).

### 2.2. Pretreatment of the Specimens

The wafers were subsequently diced into 1 × 1 cm^2^ pieces. To investigate the evolution of voids, artificial voids were formed on a flat unetched surface (referred to as the F surface) and a wet-etched surface (W surface). The detailed fabrication processes of the samples can be found in reference [25].

Before bonding, the samples were cleaned with acetone using ultrasonic waves and then dried with a N_2_ purge. After that, they were immersed in a citric acid solution, rinsed with acetone and deionized water, and dried again with an N_2_ purge.

### 2.3. Characterization of the Surface Roughness and Morphology

**Figure 1:**
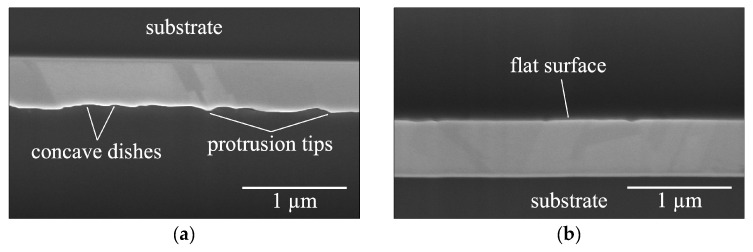
Cross-sectional SEM images of the (**a**) W surface and (**b**) F surface.

**Figure 2:**
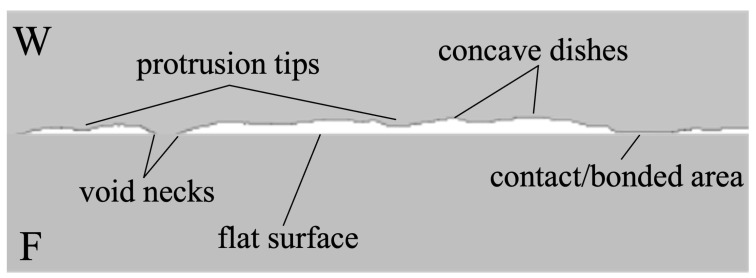
A schematic illustration of the bonded interface, based on the cross-sectional SEM images of the W and F surfaces.

Prior to bonding, Cu surface roughness was assessed by conducting atomic force microscopy (AFM, Bruker Dimension Icon Scanning Probe Microscope (ICON), Bruker, Billerica, MA, USA) measurements over a 10 × 10 µm^2^ scan area. Before and after the bonding and annealing processes, the samples were subjected to grinding and polishing. Cross-sectional specimens for scanning electron microscopy (SEM) analysis were then prepared using focused ion beam (FIB, Helios NanoLab 650, FEI, Hillsboro, OR, USA) techniques, facilitating detailed observation and characterization of the voids and interfaces in the samples. The cross-sectional SEM images of the surfaces are shown in Figure 1, revealing that the F surface appeared very flat, while the W surface exhibited protrusion tips and concave dishes. The samples were then bonded together at room temperature. A schematic illustration of the bonded interface, based on the cross-sectional SEM images of the W and F surfaces, is depicted in Figure 2.

### 2.4. Bonding Process

The samples were stacked in a fixture specifically designed for differential thermal expansion, comprising a combination of stainless steel and aluminum. This fixture was an enhanced version derived from prior research [25]. In the modified fixture, diverse stainless steel rods were utilized, namely M3 (with a major diameter of 3 mm), M4 (4 mm), and M5 (5 mm), enabling the adjustment of compressive stress levels; corresponding bonded samples were designated as M3, M4, and M5, respectively.

Initially, a minimal compressive load was applied to the sample stack at room temperature. As the temperature during processing escalated, the compressive stress on the sample stack intensified due to the disparate thermal expansion coefficients of the various materials. The resulting stresses were calculated and are presented in Table 1.

However, it is important to note that the actual stress experienced by the sample could not be accurately determined due to the occurrence of creep deformation in the Cu films at elevated temperatures. Further details regarding the deformation will be discussed in depth in the section dedicated to Cu bonding mechanism.

Artificial voids were formed at the bonded interfaces by bonding the samples for 0.5 h at 250 °C (referred to as B250t0) and 300 °C (B300t0). The bonding process took place in a vacuum environment with a pressure of 10^−3^ torr. To observe the evolution of the voids, subsequent vacuum annealing was performed at the same bonding temperature for an additional 1 h, resulting in samples denoted as B250t1 and B300t1.

## 3. Results and Discussion

**Figure 3:**
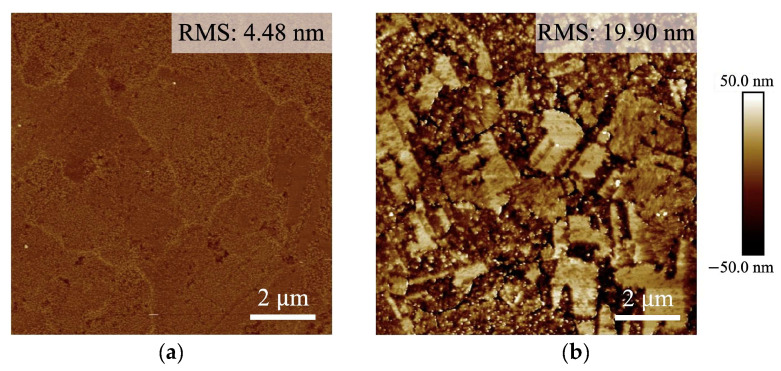
AFM topography images of the (**a**) F surface and (**b**) W surface.

The surface roughness of the Cu samples was assessed using atomic force microscopy (AFM). The root mean square (RMS) value for the F surface was determined to be 4.48 nm, while for the W surface, the RMS value measured 19.90 nm, as shown in Figure 3. After the wet-etching process, the W surface exhibited increased roughness, with a notable rise in the RMS value.

**Figure 4:**
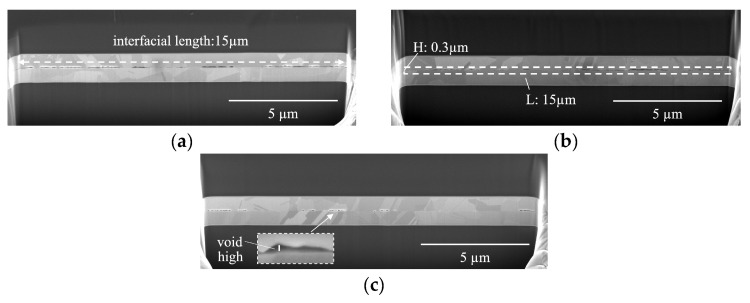
Low-magnification, cross-sectional SEM images of samples bonded at 250 °C for 0.5 h (B250t0): (**a**) M3B250t0, (**b**) M4B250t0, and (**c**) M5B250t0. The images reveal a notable increase in bonding fraction with the compressive stress. Note: The dashed arrows represent the interfacial length in (**a**). The dashed area represents the calculation range of VF in (**b**). The line represents the value of VH in (**c**).

**Figure 5:**
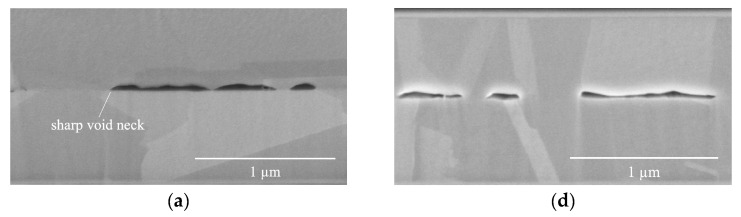

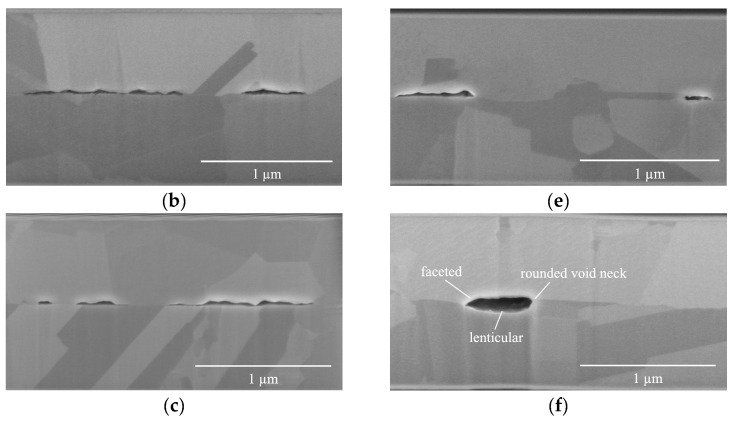
High-magnification, cross-sectional SEM images of samples bonded at 250 °C (B250): (**a**) M3B250t0, (**b**) M4B250t0, (**c**) M5B250t0, (**d**) M3B250t1, (**e**) M4B250t1, and (**f**) M5B250t1. The high-pressure conditions of the M5B250t1 bonding resulted in a significant increase in bonding fraction, followed by the closure of interfacial voids by diffusion flow, forming rounded necks and creating a lenticular shape. Note: The characteristic description of void morphology is shown in (**a**,**f**).

Figure 4 presents low-magnification, cross-sectional images of B250t0, providing an insight into the morphologies of the voids by employing bonding fraction (BF), void fraction (VF), and void height (VH) measurements. To provide a closer examination, select sections from these images have been magnified and presented in Figure 5.

BF is determined by assessing the ratio of the projected bonded/contact regions to the “interfacial length” (15 µm), as shown in Figure 4a. Void fraction (VF) is determined by comparing the void areas to the areas surrounding the interface (0.3 × 15 µm^2^), as illustrated in Figure 4b. VH represents the vertical extent of the voids, as depicted in Figure 4c.

Table 2 presents the measured values of BF, VF, and VH for the bonded interfaces of samples bonded at 250 °C (B250). It reveals a notable increase in BF with the compressive stress. The BF of M5B250t0 (${BF}_{M5B250t0}$) was 66.91%, which was higher than ${BF}_{M4B250t0}$ (43.55%) and ${BF}_{M3B250t0}$ (25.12%). Similarly, ${BF}_{M5B250t1}$ was 87.88%, which was greater than ${BF}_{M4B250t1}$ (63.82%) and ${BF}_{M3B250t1}$ (50.63%). The increase in BF can primarily be attributed to the greater creep/elastic deformation of the Cu film, which was directly influenced by the increasing compressive stress.

Two mechanisms, diffusion and deformation by yielding or creep, have been utilized to describe the morphologies of voids in Cu bonding [26]. The related deformed morphologies have been simplified as voids closed by diffusion flow to have rounded necks, and voids closed by deformation to have sharp necks, as shown in Figure 5a.

In our previous studies, we conducted extensive investigations on these two mechanisms, specifically focusing on the morphologies of void surfaces [25,27,28]. The creep deformation mechanism is primarily driven by a high stress concentration and stress gradient, while the diffusion mechanism is influenced by a reduction in surface free energy.

In the case of creep deformation, Cu atoms diffuse from the high compressive stress regions (around contact areas) towards stress-free and tensile stress regions (such as the neck, dish, and flat regions) in order to relieve stress [25,27,28]. Consequently, the deformation mechanism leads to an increase in BF and a decrease in VH. Further analysis is provided below:

In a relevant study by Juang et al. [29] on bonding in (111)-oriented nanotwinned Cu, a diffusion creep mechanism was employed. They assumed an average distance, *l*, between the center of a contacted/bonded region and an uncontacted/void region, which allowed them to determine the creep rate.

**Figure 6:**
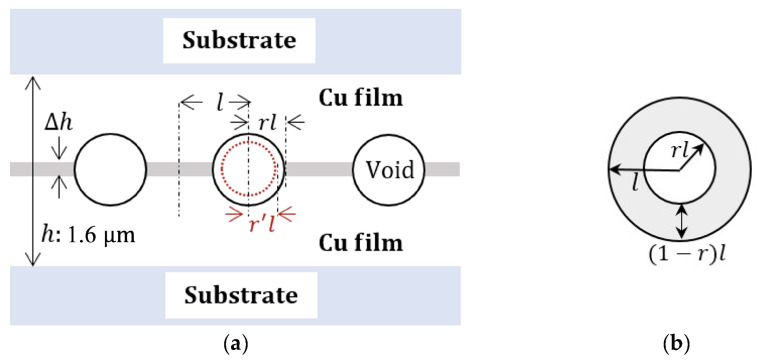
(**a**) Schematic diagram of the cross-section of part of the bonding interface. (**b**) Top view of part of the bonding interface.

In this study, a similar approach has been adopted to investigate the influence of compressive stress on the Cu bonding quality and bonding mechanism. Figure 6 illustrates the assumption made for the analysis and calculation simplification, where the void shape is assumed to be spherical with an average radius of *rl* (where *r* < 1). Based on this assumption, the bonding fraction (*BF*) can be estimated as

(1) $$BF=\frac{2l-2rl}{2l}=1-r.$$

The elastic strain, *ε*, can be calculated using the formula

(2) $$\varepsilon =\frac{\sigma }{Y}=\frac{\Delta h}{h}$$

where *σ* represents the uniform compressive stress, *Y* is the Young’s modulus of Cu, Δ*h* represents the change in thin film thickness under compression, and *h* represents the total thickness of the Cu film (1.6 μm).

According to Table 1, at 250 °C, the calculated compressive stress of M5 ($\sigma_{M5}$) was 53.64 MPa, which was greater than $\sigma_{M4}$ (41.77 MPa) and $\sigma_{M3}$ (29.17 MPa). As a result, $\Delta h_{M5}$ would be greater than $\Delta h_{M4}$ and $\Delta h_{M3}$ due to the higher compressive stress level.

In order to examine the impact of compressive stress on the bonding fraction (BF), two volumes are considered: the strained volume ($V_{strained}$) and the reduced void volume (${∆V}_{void}$). The objective of creep deformation is to relocate all the atoms within the strained volume ($V_{strained}$) from the bonded region to the void region [29]. As shown in Figure 6, the strained volume can be approximated as

(3) $$V_{strained}=A\Delta h$$

where *A* represents the contacted area given by

(4) $$A={[l}^{2}-{\left(rl\right)}^{2}]\pi .$$

Thus, the strained volume can be expressed as

(5) $$V_{strained}=[1-r^{2}]\pi l^{2}\Delta h.$$

Additionally, the reduced void volume (${∆V}_{void}$) can be determined by considering the change in void radius resulting from creep deformation. It can be calculated using the formula

(6) $${∆V}_{void}=\frac{4\pi \left[{\left(lr\right)}^{3}-{\left(lr^{\prime }\right)}^{3}\right]}{3}$$

where $lr^{\prime }$ represents the new void radius after the creep deformation takes place.

When we equate the two expressions of volume, we obtain

(7) $$\left[1-r^{2}\right]\pi l^{2}\Delta h=\frac{4\pi \left[{\left(lr\right)}^{3}-{\left(lr^{\prime }\right)}^{3}\right]}{3}$$

(8) $${\left(r^{\prime }\right)}^{3}=r^{3}-\frac{3\left[1-r^{2}\right]\Delta h}{4l}.$$

This calculation suggests that as Δh increases, $r^{\prime }$ decreases. Since $\Delta h_{M5}$ was greater than $\Delta h_{M4}$ and $\Delta h_{M3}$, $r_{M5}^{\prime }$ was smaller than $r_{M4}^{\prime }$ and $r_{M3}^{\prime }$. As mentioned earlier, BF is calculated as 1 *−* $r^{\prime }$, which means that BF increases as $r^{\prime }$ decreases. Consequently, ${BF}_{M5}$ was greater than ${BF}_{M4}$ and ${BF}_{M3}$.

**Figure 7:**
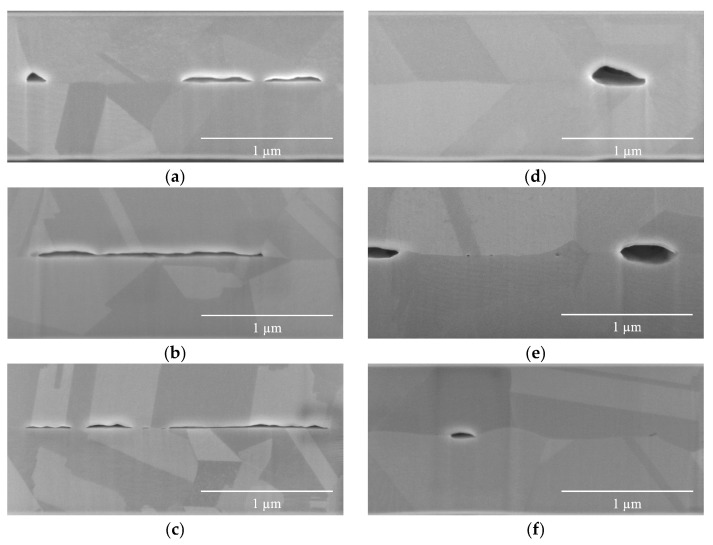
Cross-sectional SEM images of samples bonded at 300 °C (B300): (**a**) M3B300t0, (**b**) M4B300t0, (**c**) M5B300t0, (**d**) M3B300t1, (**e**) M4B300t1, and (**f**) M5B300t1. Under high-pressure bonding conditions, the void height (VH) decreases as the bonding fraction (BF) increases, leading to a decrease in the influence of creep deformation, and the dominant mechanism transitions to diffusion, which results in the formation of lenticular shapes.

It can be concluded that the same trend applies to samples bonded at 300 °C, as seen in Table 3 and Figure 7. The BF of M5B300t0 (${BF}_{M5B300t0}$) was 71.84%, which was higher than ${BF}_{M4B300t0}$ (68.43%) and ${BF}_{M3B300t0}$ (60.9%). Similarly, ${BF}_{M5B300t1}$ was 94.16%, surpassing both ${BF}_{M4B300t1}$ (88.81%) and ${BF}_{M3B300t1}$ (88.51%).

Table 2 also indicates that the VF decreased as the compressive stress increased. For instance, the VF of the M5B250t0 sample (${VF}_{M5B250t0}$) was 1.99%, which was lower than ${VF}_{M4B250t0}$ (4.57%) and ${VF}_{M3B250t0}$ (5.53%). Similarly, ${VF}_{M5B250t1}$ was 1.54%, which was lower than ${VF}_{M4B250t1}$ (2.89%) and ${VF}_{M3B250t1}$ (4.17%). This observation remains valid for samples bonded at 300 °C (B300), as evidenced by the data presented in Table 3. The decrease in VF can be primarily explained by the corresponding increase in both creep deformation and BF as the compressive stress increases. Further analysis is provided below.

As mentioned earlier, the void fraction (*VF*) was determined by calculating the ratio of the void areas to the areas surrounding the interface. According to Figure 6, the *VF* estimation is adjusted using the equation

(9) $$VF=\frac{\pi {\left(lr^{\prime }\right)}^{2}}{2lH}$$

where *H* = 0.3 µm represents the height of the areas surrounding the interface, as illustrated in Figure 4b. It can be observed that *VF* increases as ${\left(r^{\prime }\right)}^{2}$ increases, which in turn happens when Δh decreases. In other words, *VF* increases as the compressive stress decreases.

The changes in VH with varying compressive stress were influenced by the bonding mechanism and the bonding fraction (BF). Figure 5a–c display the morphologies of void necks in the B250t0 samples, exhibiting a relatively sharp shape. This observation suggests that the primary bonding mechanism in the B250t0 samples was creep deformation. As shown in Table 2, the VF of the M5B250t0 sample (${VH}_{M5B250t0}$) was 41.2–63.5 nm, which was lower than ${VH}_{M4B250t0}$ (22.2–66.6 nm) and ${VH}_{M3B250t0}$ (25.4–95.2 nm). The observed findings suggest a correlation between a decrease in void height (VH) and an increase in compressive stress. This relationship can be attributed to the phenomenon of creep deformation, where Cu atoms diffuse from the contact areas towards the neck, dish, and flat regions in order to alleviate stress [25,27,28]. As a result, this deformation mechanism ultimately leads to a decrease in VH. Further analysis is provided below.

Referring to Figure 6, the estimation of *VH* is adjusted using the equation

(10) $$VH=2lr^{\prime }$$

where $lr^{\prime }$ represents the new void radius. As mentioned earlier, $r_{M5}^{\prime }$ was smaller than $r_{M4}^{\prime }$ and $r_{M3}^{\prime }$; therefore, ${VH}_{M5B250t0}$ was smaller than ${VH}_{M4B250t0}$ and ${VH}_{M3B250t0}$.

This observation holds true for ${VH}_{M3B250t1}$ and ${VH}_{M4B250t1}$ as well. The bonding mechanism in these two cases was primarily governed by deformation. As indicated by the data presented in Table 2, ${VH}_{M3B250t1}$ was 44.4–88.8 nm, which was higher than ${VH}_{M4B250t1}$ (24.4–57.5 nm). However, ${VH}_{M5B250t1}$ (44.4–158.6 nm) was higher than ${VH}_{M4B250t1}$ and ${VH}_{M3B250t1}$. This can be attributed to a change in the bonding mechanism to diffusion, as evidenced by the rounded neck and lenticular shape of M5B250t1 voids depicted in Figure 5f.

As mentioned earlier, the diffusion mechanism occurs through a reduction in surface free energy. As depicted in Figure 2, the morphologies of void surfaces are associated with four types of free energies: free energy at the protrusion tip (*G_+tip_*), free energy at the flat surface (*G_flat_*), free energy at the concave dishing (*G_-dish_*), and free energy at the void neck (*G_-neck_*). The diffusion of Cu atoms from protrusion tips to dishing regions and void necks leads to a reduction in free energy, ultimately resulting in an increase in VH [27].

Furthermore, as Cu atoms diffuse from the flat surface (F surface) towards void necks, certain F surfaces experience a change in their radius of curvature (*R_flat_*) from infinite to negative or faceted, thus forming lenticular and faceted voids, as shown in Figure 5f. These observations align with the findings of Gondcharton et al. [30], who conducted a study on Cu–Cu bonded structures. This diffusion process occurring at the “F surface” also contributes to the observed increase in VH.

This transition of the dominant bonding mechanism can be attributed to the increase in bonding fraction (BF). This change can be understood by considering the atomic creep flux, as elucidated by Juang et al. [29] in their study on the bonding of nanotwinned Cu. The flux can be represented by the equation [31]

(11) $$J=\frac{D\sigma_{contact}}{kTl}$$

where *J* is the creep flux, *D* is the diffusivity of Cu, $\sigma_{contact}$ is the actual stress at the contact area, *k* is Boltzmann’s constant, *T* is the temperature, and *l* represents the average distance between the center of a bonded region and a void region (as illustrated in Figure 6).

The stress at the contacted area can be estimated as

(12) $$\sigma_{contact}\approx \frac{C\sigma }{BF}$$

where *C* is a proportionality constant and *σ* is the uniform compressive stress, as mentioned earlier. This relationship indicates that as the bonding fraction (*BF*) increases, the actual stress at the contacted area ($\sigma_{contact}$) decreases. This implies that the influence of creep deformation decreases with an increase in BF. When the bonding fraction is high, the contribution of creep deformation to the overall bonding mechanism diminishes, and the dominant mechanism transitions to diffusion. As mentioned earlier, ${BF}_{M5B250t1}$ was 87.77%, which was greater than ${BF}_{M4B250t1}$ (63.82%) and ${BF}_{M3B250t1}$ (50.63%).

This conclusion applies equally to both the B300t0 and B300t1 groups of specimens. Regardless of the specific group, as the bonding fraction (BF) increases, the influence of creep deformation gradually weakens, and the diffusion mechanism becomes the dominant bonding mechanism. The observations from Table 3 and Figure 7 support the notion that in B300t0 specimens, the dominant bonding mechanism is deformation, while in B300t1 specimens, diffusion becomes the dominant mechanism.

## 4. Conclusions

Artificial voids were introduced at the bonded interfaces to investigate the influence of compressive stress on the bonding quality and bonding mechanisms at elevated temperatures of 250 °C and 300 °C. We compared three different levels of compressive stress in Cu–Cu bonding and found that as the compressive stress increases, there is an increase in the bonding fraction (BF) and a decrease in the void fraction (VF). This result indicates that high compressive stress is beneficial for promoting interface healing and enhancing bonding quality. Furthermore, these experimental findings are supported by the surface diffusion creep model.

This study offers direct evidence of the correlation between compressive stress and the quality of the bonding interface. Ensuring high-quality Cu–Cu bonding interfaces requires careful consideration of the compressive stress during the bonding process. In the future, we aim to validate these findings further through hybrid bonding, which we anticipate will offer significant advantages for 3D package applications.

## Figures and Tables

**Table 1:** The calculated compressive stresses at 250 °C and 300 °C.

	M3	M4	M5
250 °C	29.17 MPa	41.77 MPa	53.64 MPa
300 °C	35.65 MPa	51.06 MPa	65.56 MPa

**Table 2:** The measured bonding fraction (BF), void fraction (VF), and void height (VH) of samples bonded at 250 °C (B250).

	M3B250t0	M4B250t0	M5B250t0	M3B250t1	M4B250t1	M5B250t1
BF (%)	25.12	43.55	66.91	50.63	63.82	87.77
VF (%)	5.53	4.57	1.99	4.17	2.89	1.54
VH (nm)	25.4–95.2	22.2–66.6	41.2–63.5	44.4–88.8	24.4–57.5	44.4–158.6

**Table 3:** The measured bonding fraction (BF), void fraction (VF), and void height (VH) of samples bonded at 300 °C (B300).

	M3B300t0	M4B300t0	M5B300t0	M3B300t1	M4B300t1	M5B300t1
BF (%)	60.9	68.43	71.84	88.51	88.81	94.16
VF (%)	3.16	2.97	1.61	1.85	1.84	1.09
VH (nm)	50.8–114.2	34.9–60.3	28.6–47.6	82.5–209.4	120.6–222.1	63.5–209.4

## Data Availability

Data are contained within the article.
